# Circulating Tumor DNA in Identifying Resistant Sub-Clones Post EGFR Blockade: Implications for EGFR Rechallenge

**DOI:** 10.3389/fonc.2022.847299

**Published:** 2022-06-28

**Authors:** Adithya Chennamadhavuni, Pashtoon Murtaza Kasi

**Affiliations:** ^1^ Holden Comprehensive Cancer Center, University of Iowa Hospitals and Clinics, Iowa City, IA, United States; ^2^ Englander Institute for Precision Medicine, Weill Cornell Medical College, New York, NY, United States

**Keywords:** anti-EGFR therapy, ctDNA, rechallenge, metastatic colorectal cancer, cetuximab, panitumumab, tumor heterogeneity, evolution

## Abstract

For patients with metastatic *RAS/RAF* wild-type refractory colorectal cancer, the question of anti-EGFR therapy rechallenge often comes up after initial use. However, not all patients derive benefit. It is now well known that these tumors acquire mechanisms of resistance in the mitogen-activated protein kinase (MAPK) pathway, which can be detected on circulating tumor DNA (ctDNA)-based testing. We present a series of patients who had serial testing post-EGFR blockade showing its feasibility and value. This would have implications for EGFR rechallenge. We reviewed records for patients who were initially noted to be *RAS/RAF* wild-type on tissue, who received prior anti-EGFR therapy and then subsequently had at least one circulating tumor DNA-based testing. These patients also had tissue-based genomic testing obtained earlier as part of their standard of care, alongside serial ctDNA-based testing that was done later when subsequent lines of therapy were being decided. The median duration of initial prior anti-EGFR therapy was around 10 months. Known acquired mechanisms of resistance were noted in 100% of the cases. These included *KRAS*, *NRAS*, extracellular domain mutations in *EGFR*, and *BRAF* mutations. Interestingly, the levels of the sub-clones expressed in variant allele fraction percentage varied and decreased over time in relation to timing of the prior EGFR exposure. Additionally, these were noted to be polyclonal, and the number of clones also varied including some disappearing over time during non-EGFR-based therapy (EGFR holiday). Patients’ post-EGFR blockade may have multiple mechanisms of acquired resistance that can be easily detected on non-invasive liquid biopsies. These patients do not benefit from EGFR rechallenge based on the results of the recently reported CRICKET (NCT02296203) and CAVE (NCT04561336) clinical trials. Furthermore, excluding these patients from EGFR rechallenge is already being adopted in prospectively done clinical trials, e.g., the CHRONOS study (NCT03227926). Rechecking the liquid biopsy plasma *RAS/RAF* status is one thing that may be incorporated into practice with EGFR rechallenge only a consideration if acquired mechanisms of resistance are absent.

## Introduction

Advanced colorectal cancer (CRC) patients with a *RAS/RAF* wild-type status obtain significant benefits from anti-epidermal growth factor receptor antibody (anti-EGFR) therapy in combination with chemotherapy (1–5). Sidedness plays an important role with its approval as first-line therapy only in left-sided tumors, and with subsequent lines among the right-sided tumors (6). Unfortunately, like any targeted therapy, these tumors develop secondary acquired mechanisms of resistance. For patients with metastatic *RAS/RAF* wild-type refractory colorectal cancer, the question of anti-EGFR therapy rechallenge often comes up after initial use. However, not all patients derive benefit. It is now well known that these tumors acquire mechanisms of resistance in the mitogen-activated protein kinase (MAPK) pathway, which can be detected on circulating tumor DNA (ctDNA)-based testing (6, 7).

**Figure 1:**
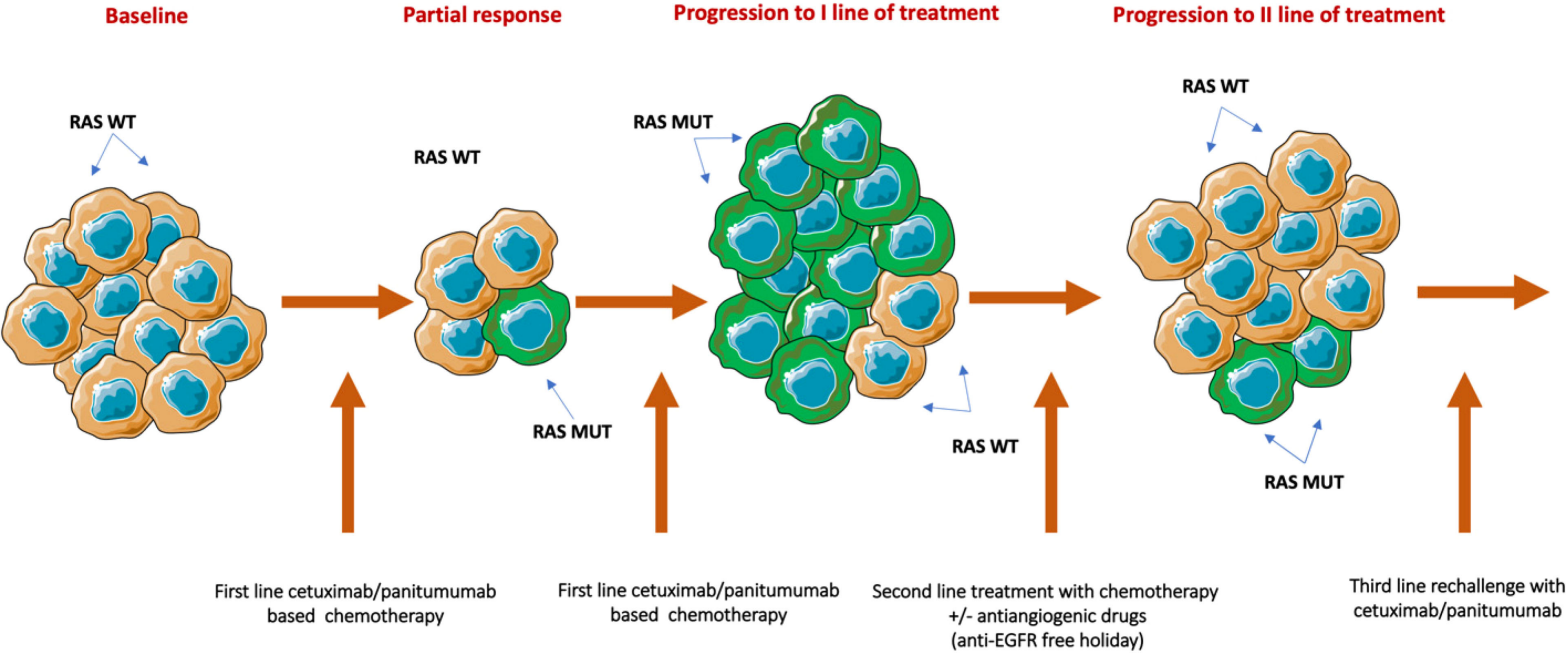
Biological rationale for rechallenge therapy. Treatment with anti-EGFR inhibitors rapidly eliminates RAS WT-sensitive clones and favors the expiation of resistant cancer cells. After disease progression, and due to the administration of a second line of chemotherapy without anti-EGFR monoclonal antibodies, RAS mutant clones progressively decay, inducing the proliferation of RAS WT cell. WT, Wild type; MUT, Mutant;/: Or. [Published with permission from Cancers 2021, 13(8), 1941: open access (11)].

Solid tumors change over time and space from clonal evolution, causing significant intra-tumor genetic heterogeneity, contributing resistance to chemotherapy and biologics. When patients have disease progression after first-line combination chemotherapy and anti-EGFR therapy, knowing the mechanism(s) of resistance can be important. Patients who developed resistance to chemotherapy can continue with anti-EGFR therapy with a change in the chemotherapy backbone (8–10). Eventually, patients develop resistance to targeted therapy from selection pressure resulting in disease progression. When patients have a break from targeted therapy, tumors can potentially get resensitized to anti-EGFR therapy by a reduction in clonal selection pressure, as depicted in **Figure 1**. These patients would potentially derive more benefit from targeted therapy, rather than the broad rechallenge among all patients who are progressing. Rechallenge with anti-EGFR therapy in combination with multiple therapies was evaluated in several retrospective and prospective studies (12–16).

Tissue biopsy to identify the clonal status in the tumor is limited by the procedural risk and availability of enough tissue. Liquid biopsies are gaining importance in capturing tumor heterogeneity, negating the procedural risk. The testing involves circulating or cell-free DNA testing in the circulation, represented by circulating tumor DNA when the origin is from tumor tissue. Liquid biopsy may better assess tumor heterogeneity as ctDNA is released into circulation by the primary tumor and the multiple metastatic sites. Among colorectal cancer patients, various studies have shown that above 20% (17) have difficulty obtaining tissue for molecular analysis, while 15% of patients had no shedding of ctDNA (18–20). Negative ctDNA could be secondary to low tumor volume or the timing of testing, such as post-chemotherapy or postoperative periods. The reliability of liquid biopsy to determine tumor evolution is depicted in the recent CAVE (14), CRICKET (13), and CHRONOS clinical trials (15) and is currently under evaluation in REMARRY/PURSUIT trials (21).

We present a series of patients who had serial testing post EGFR blockade showing its feasibility and value. This would have implications for EGFR rechallenge.

## Materials and Methods

This is a retrospective study. After the approval from the Institutional Review Board (IRB), records for patients who were initially noted to be *RAS/RAF* wild-type on tissue eligible to receive anti-EGFR therapy from 2019 to 2021 were reviewed. We used two commercially available liquid biopsy circulating tumor DNA (ctDNA) platforms that are next-generation sequencing (NGS) based. The choice of the assay was more so due to institutional preference and/or insurance coverage for one assay or the other. Both Tempus xF and Guardant360 assays are CLIA approved. The Guardant360 assay is also US FDA approved. Both assays have numerous validity, concordance, and studies relating to sensitivity and specificity of these assays.

There is little concern regarding the ability of these ctDNA-based platforms with respect to detecting mutations. They are both based on hybrid capture NGS testing, which facilitates broader gene sequencing than hot spot-based NGS amplification with an extensive repertoire for detecting aberrations. Fusions are large gene rearrangements that can also be seen post EGFR challenge. When detected, it is highly specific for these aberrations (22). However, absence of this on the reports does not rule out the presence of fusions (23). This work pertains to more so mutations that were detected post EGFR challenge.

### Sequencing and Analysis

G360 (Guardant Health, Redwood City, CA, USA) is a commercially available 74-gene panel plasma-based tumor genomic profiling assay validated to detect a variety of genomic alterations including MSI-H signature (24), single-nucleotide variants, indels, and copy number alterations (amplifications and fusions) (23) in cell-free DNA (cfDNA) from plasma of patients with solid tumors, including colorectal cancer.

The Tempus xF assay (25) assay detects SNVs and indels in 105 genes, CNVs in 8 genes, and chromosomal rearrangements/fusions for 7 genes by DNA-Seq. The average depth of coverage is 5,000 unique reads per 20,000 raw reads.

Patients were followed with ctDNA at multiple variable time points while on therapy for clonal evolution of resistance to anti-EGFR therapy. The resistance was determined through the appearance of mutations in the MAPK pathway (*RAS*, *RAF*, and *EGFR* domain mutations), which are not present during prior ctDNA testing.

## Results

Upon reviewing, we found six patients who met the inclusion criteria. **Table 1** shows results of the patients’ tissue-based genomic testing in parallel with the serial circulating tumor DNA-based testing that was done during the time of follow-up scans and/or when subsequent lines of therapy were being decided. The median duration of anti-EGFR therapy was around 10 months. Resistance to anti-EGFR therapy is by constitutive activation of EGFR downstream signaling pathways regardless of EGFR blockade predominantly through *KRAS*, *NRAS*, *BRAF*, and extracellular domain mutations in *EGFR* (26). HER2/*ERBB2* aberrations were also noted. As noted in **Table 1** the known acquired mechanisms of resistance were noted in 100% of the cases. Interestingly, the levels of the sub-clones expressed in variant allele fraction percentage varied and decreased over time in relation to the timing of the prior EGFR exposure. Additionally, these were noted to be polyclonal, and the number of clones also varied over time. Some clones disappeared over time during the non-EGFR-based therapy (EGFR holiday such as *KRAS* clones in patient 2, *BRAF* clones in patient 3, and *EGFR* clones in patient 4. Anti-EGFR therapy was only attempted in patients who had no known RAS/RAF resistance clones, which did lead to a response lasting 4 months (8 cycles) in 2 of these 6 patients.

**Table 1:** – *+ indicates the timepoint where the named clones were detected.

	APC	TP53	KRAS	NRAS	BRAF	EGFR
Patient 1						
Tissue NGS	+	+				
ctDNA – T1*	+	+	+		+	+
ctDNA – T2	+	+	+			+
ctDNA – T3	+	+	+		+	+
Patient 2						
Tissue NGS	+	+				
ctDNA – T1*	+	+				
ctDNA – T2	+	+				
ctDNA – T3						
ctDNA – T4	+	+	+			
ctDNA – T5						
Patient 3						
Tissue NGS	+	+				
ctDNA – T1*	+	+				
ctDNA – T2*	+	+			+	+
ctDNA – T3	+	+	+			+
ctDNA – T4	+		+			+
ctDNA – T5	+	+	+			+
Patient 4						
Tissue NGS	+	+				
ctDNA – T1*	+	+	+			
ctDNA – T2	+	+	+			+
ctDNA – T3	+	+	+		+	+
ctDNA – T4	+	+	+	+	+	
Patient 5						
Tissue NGS	+	+				
ctDNA – T1*	+	+				+
ctDNA – T2	+	+				+
ctDNA – T3	+	+				+
Patient 6						
Tissue NGS	+	+				
ctDNA – T1*	+	+	+		+	
ctDNA – T2	+	+	+		+	
ctDNA – T3	+	+	+		+	

Known acquired mechanisms of resistance were noted in all the cases. The sub-clones were noted to be polyclonal, and the number of clones varied over time. Some clones disappeared over time during non-EGFR-based therapy (EGFR holiday such as KRAS clones in patient 2, BRAF clones in patient 3, and EGFR clones in patient 4.

## Discussion

Precision medicine and utilization of targeted therapy are increasingly gaining importance in the modern therapeutic landscape. ctDNA is gaining significant momentum in aiding important clinical decisions in utilization of the targeted therapies. An earlier study by Ciardiello et al. from the CAPRI-GOIM trial included tissue-based NGS testing to identify patients without downstream mutations depicting benefit with anti-EGFR therapies (27). The combination therapy showed an objective response rate (ORR) of 62.0% (95% CI 55.5%–74.6%) with a median progression-free survival (mPFS) of 11.1 (95% CI 9.2–12.8) months in patients with *KRAS* and *NRAS* wild-type tumors. Patients with *KRAS* or *NRAS* mutations had a significantly lower ORR of 46.6% (95% CI 39.9–57.5%) with mPFS of 8.9 (95% CI 7.4–9.6) months. Further, retrospectively designed studies show that ctDNA detected *KRAS* and/or *EGFR* mutations in patients unresponsive to anti-EGFR treatment (19, 28). The presence of such clones was associated with shorter PFS in comparison with no mutations (3 versus 8 months, P = 0.0004). There were multiple simultaneous mutations in *KRAS* and *EGFR* in the ctDNA with a decrease in decline in variable allele frequency (VAF) after stopping the therapy (19). Our study shows a similar evolution of resistance clones with a decline in their VAF by following these patients through their course of anti-EGFR therapy, building further confidence in utilization of ctDNA, a reliable marker for day-to-day clinical decision making (12).

At present, there are several prospective studies that have reported on the importance of determining the *RAS/RAF* status on the liquid/plasma determining response to targeted therapies. The first landmark observations came from the CRICKET and CAVE clinical trials (29, 30), which both showed a lack of benefit of anti-EGFR therapy in patients who had these acquired mechanisms of resistance detected in plasma. These led to a recent study called CHRONOS that just got reported out and presented at the American Society of Clinical Oncology (ASCO) meeting in June 2021, whereby patients were actually excluded if they had their resistance mechanisms detected in the blood. This led to a clinically meaningful ORR of 30% (95% CI: 12%–47%), a DCR (PR plus SD > 4 months) of 59% (95% CI: 41%–78%), and an mPFS of 16 weeks. Large retrospective series demonstrated ctDNA utilization in detecting NTRK1 (31), RET, and FGFR3 fusions (22) apart from subclonal RAS and EGFR mutations which were hypothesized to contribute to anti-EGFR resistance (32). Such mechanisms need to be studied in prospective fashion to determine treatment implications with specific targeted agents.

Our study reports on the real-time utility and feasibility of incorporating the evaluation of ctDNA liquid *RAS/RAF/EGFR* and the status of other relevant resistance mutations in blood in patients with prior anti-EGFR exposure and tissue RAS/RAF-wild-type tumor. Given the lack of benefit of anti-EGFR therapy in patients whose liquid biopsies reveal known resistance mechanisms to these drugs, it makes sense not to use these drugs in those patients at that point in time. Given that there is exponential decay that can happen potentially on EGFR holiday, the liquid RAS/RAF/EGFR status could later be rechecked to guide the optimal timing of EGFR rechallenge.

## Conclusion

In summary, here we report on a case series illustrating the feasibility of obtaining ctDNA in real time to assess for the presence or absence of acquired resistance mutations. These were present in 100% of the patients initially, with decay over time allowing for rechallenge. With several clinical trials now reporting on the lack of benefit in patients who have these resistance mutations, assessment of this prior to EGFR rechallenge would be a consideration to include in guidelines.

## Data Availability Statement

The original contributions presented in the study are included in the article/supplementary material. Further inquiries can be directed to the corresponding author.

## Ethics Statement

The studies involving human participants were reviewed and approved by the University of Iowa Institutional Review Board. The patients/participants provided their written informed consent to participate in this study.

## Author Contributions

The authors confirm contribution to the paper as follows: study conception and design: PK; data collection: AC; analysis and interpretation of results: AC; draft manuscript preparation: AC and PK. All authors contributed to the article and approved the submitted version.

## Conflict of Interest

Author PMK: Consulting/Advisory Board: Merck/MSD, Servier, Delcath, Foundation Medicine, Taiho (self/institution), Tempus, QED, Eli Lilly, Daiiche Sankyo, Bayer, Incyte, Eisai, Natera, Exact Sciences, Ipsen (institution). Research support to institution: BTG/Boston Scientific, AstraZeneca, Tersera, RenovoRX.

The remaining author declares that the research was conducted in the absence of any commercial or financial relationships that could be construed as a potential conflict of interest.

## Publisher’s Note

All claims expressed in this article are solely those of the authors and do not necessarily represent those of their affiliated organizations, or those of the publisher, the editors and the reviewers. Any product that may be evaluated in this article, or claim that may be made by its manufacturer, is not guaranteed or endorsed by the publisher.
